# Optimizing Antiretroviral Therapy in Heavily ART-Experienced Patients with Multi-Class Resistant HIV-1 Using Proviral DNA Genotypic Resistance Testing

**DOI:** 10.3390/v15071444

**Published:** 2023-06-27

**Authors:** Dominic Rauschning, Ira Ehren, Eva Heger, Elena Knops, Gerd Fätkenheuer, Isabelle Suárez, Clara Lehmann

**Affiliations:** 1 Division of Infectious Diseases, Department I of Internal Medicine, Medical Faculty and University Hospital Cologne, University of Cologne, 50937 Cologne, Germany; 2 Department Ib of Internal Medicine, Bundeswehrzentralkrankenhaus Koblenz, Rübenacher Straße 170, 56072 Koblenz, Germany; 3 Institute of Virology, University of Cologne, Faculty of Medicine and University Hospital Cologne, Fürst-Pückler-Straße 56, 50935 Cologne, Germany; 4 Center for Molecular Medicine Cologne (CMMC), Medical Faculty and University Hospital Cologne, University of Cologne, 50937 Cologne, Germany; 5 Partner Site Bonn-Cologne, German Center for Infection Research (DZIF), 38124 Braunschweig, Germany

**Keywords:** HIV infection, multi-class resistance, antiretroviral therapy, therapy adjustment, diagnostic tests, resistance testing, proviral DNA

## Abstract

Resistance to multiple antiretroviral drugs among people living with HIV (PLWH) can result in a high pill burden, causing toxicity and drug interactions. Thus, the goal is to simplify treatment regimens while maintaining effectiveness. However, former resistance analysis data may not be current or complete. The use of proviral DNA genotyping may assist in selecting appropriate treatment options. A retrospective study was carried out on individuals belonging to the Cologne HIV cohort with a resistance history to two or more antiretroviral (ARV) classes and on non-standard antiretroviral therapy (ART). Patients required former viral RNA and a recent proviral DNA resistance test to be available prior to the switch to ART. Potential discrepancies between resistance test results obtained through RNA and proviral DNA methods and the consequent virological and clinical outcomes following ART adjustments were analyzed. Out of 1250 patients, 35 were eligible for inclusion in this study. The median length of known HIV infection was 27 years, and the median duration of ART was 22 years. Of the 35 participants, 16 had received all five ARV classes. Based on proviral DNA genotyping results, ART was simplified in 17 patients. At the last follow-up examination after changing therapy, 15 patients had HIV RNA <50 copies/mL (median 202 days, range 21–636). The mean number of pills per day decreased from eight to three, and the median intake frequency decreased from two to one time/day (ranges 1–2). Our study supports the use of proviral DNA genotyping as a safe strategy for switching to simplified ART regimens. However, the lack of extensive research on the advantages of proviral DNA genotyping makes it challenging to fully assess its benefits in terms of treatment selection.

## 1. Introduction

With the progress made in antiretroviral therapy (ART), people living with HIV (PLWH) have been able to achieve life expectancies that are similar to those of non-infected people in Europe and North America [1]. Improved therapy has led to a decline in the prevalence of multi-class resistances, which currently play a minor role in contrast to the beginning of the epidemic [2,3]. The continued development of newer antiretroviral drugs (ARVs) has led to higher resistance barriers, greater effectiveness and new mechanisms of action. This has also reduced the likelihood of adverse effects, drug interactions and toxicity. Furthermore, the optimization of formulations, enabling drug combinations as single-tablet regimens, has simplified therapy modality with virus control, ultimately leading to an improvement in the quality of life of PLWH on therapy [4]. Nevertheless, a few individuals, mostly infected before the introduction of potent ART in 1996, have developed extensive resistance to ARV classes, often due to treatment failure based on the low resistance barriers of previous active drugs, a lack of pharmacological combination partners, but also a lack of adherence. This necessitates intensive therapeutic intervention using non-standard combinations of substances and, in some cases, with a variety of substances, leading to comprehensive ART. It also led to a high pill burden and a higher probability of drug interactions and adverse drug effects [5,6]. These long-term survivors are now reaching an age where complex chronic diseases such as diabetes mellitus or coronary heart disease with chronic heart failure are making therapies even more complex. HIV treatment providers are facing the question of how to switch these patients to simplified modern ART regimens without losing infection control and inducing further resistance. One challenge in this situation is the effective use of resistance analysis, which were mostly conducted a long time ago as they were typically carried out before therapy initiation or in the case of therapy failure [7]. This can pose difficulties since the information gathered during resistance analysis may be outdated or incomplete or not accurately reflect the patient’s current condition. Resistance testing using proviral deoxyribonucleic acid (DNA), which conserves virus variants and drug resistance mutations (DRM) [8,9] in the cells of the HIV reservoir, even under maximally suppressed viral loads [8,10,11], can provide valuable information on current mutations and help make informed therapy decisions.

Understanding HIV-1 dynamics in PBMC, particularly in patients with suppressed plasma viremia, is important for the optimal use of proviral DNA resistance analysis in clinical decision-making. In acute infection, proviral DNA levels and diversity increase within the initial years [12]. Without ART, proviral DNA reaches an equilibrium between processes that enhances and diminishes its presence. CD4+ lymphocytes become infected during active virus replication and latent cell proliferation, leading to increased proviral DNA levels [13]. Thus, untreated patients experience continuous evolution of the proviral DNA reservoir [14]. Following the initiation of ART, the turnover of the proviral DNA reservoir decelerates. In untreated patients, the estimated half-life of this compartment is around 1 year, whereas, in virologically suppressed patients, it extends to approximately 3 to 4 years [15]. This reduced turnover rate is attributed to the gradual increase in clonal proliferation of latently infected cells over time [16]. The objective of the study was to evaluate both clinical and virological outcomes after the simplification of ART in patients with intensive ARV treatment based on proviral DNA resistance testing. Additionally, the study aimed to compare the resistance test results obtained through ribonucleic acid (RNA) and proviral DNA methods and assess any potential discrepancies between them [17,18].

## 2. Materials and Methods

### 2.1. Study Design and Population

The study was a retrospective longitudinal cohort analysis of individuals living with long-term HIV-1 infection who were on non-standard antiretroviral therapy (ART) due to previous resistance to two or more classes of antiretroviral drugs. Standard ART was defined as therapy utilizing a Nucleoside Reverse Transcriptase Inhibitor (NRTI) backbone as well as either a Non-Nucleoside Reverse Transcriptase Inhibitor (NNRTI) or an integrase inhibitor (INI) or a (boosted) protease inhibitor (PI), in accordance with 2019 WHO therapy guidelines [19]. The analyzed patients were required to have undergone a resistance test from both viral RNA and proviral DNA. The analysis took into account all resistances of a patient that were ever detected in genotypic resistance tests of viral RNA. Only the most recent genotypic resistance test of proviral DNA was used if several were available. Drug changes were initiated by either the practitioner or the patient’s request to simplify therapy, specifically by reducing the number of tablets and potential side effects due to drug–drug-interactions. Subsequently, patients were selected from the cohort whose therapy could be simplified based on the results from proviral resistance testing. The clinical and virological course of these patients was then further evaluated in the subsequent analyses. Data from 15 April 1985 to 19 September 2021, including demographic, epidemiological, and therapy information, were collected and are presented in Table 1 and Table 2. For patients with therapy adjustment, additional information on HIV viral load, tablets count, frequency of medication administration per day and ARV regimen before and after therapy adjustment were obtained.

### 2.2. Resistance Analysis

Genotypic resistance testing of viral RNA was performed using Sanger sequencing with ViroSeq HIV genotyping kit v2.0 (Applied Biosystems, Waltham, MA, USA) until the end of 2014 or with new-generation sequencing (NGS) with MiSeq (Illumina, San Diego, CA, USA) afterwards. Both platforms have the capability to accurately sequence and identify DRMs in peripheral viral loads >1000 cop/mL. The MiSeq platform exhibits excellent levels of sensitivity and specificity in identifying DRMs that are also detectable through Sanger sequencing [20,21]. For genotypic resistance testing of proviral DNA, only NGS with MiSeq (Illumina, San Diego, CA, USA) was used. Hypermutations induced by apolipoprotein B mRNA editing enzyme catalytic polypeptide 3 (APOBEC3) were removed using bioinformatic filters. The cut-off for the resistance mutations sensitivity to also detect minor virus strains was 2%, as used in the daily clinical routine of the Institute of Virology. The Stanford HIV Drug Resistance Database was used to interpret ART resistance [22]. To compare resistance tests of viral RNA and proviral DNA, the number of genotypic resistance tests of viral RNA and proviral DNA and the time between resistance tests and the number of resistance mutations detected in viral RNA and DNA were used. For patients who received a treatment simplification based on the results of proviral DNA resistance testing, the course of the HIV RNA before and after the adjustment was compared.

### 2.3. Statistical Analysis

Participant characteristics were examined using descriptive statistics, such as mean, median and relative frequency. When comparing the pill burden, decimal places were rounded up to the next integer value. Differences between the results were tested for significance using the paired t-test for means (if normal distribution) and using the Wilcoxon signed rank test (if non-normal distribution) for medians. Statistical significance was defined as a two-tailed *p*-value < 0.05. The statistical analysis was carried out with the statistics program Sigma Plot 2.5 for Windows.

### 2.4. Ethics

The study was conducted at the University Hospital of Cologne and approved by the ethics committee of the medical faculty (reference: 20-1484).

## 3. Results

### 3.1. Characteristics of the Patient Group

**Figure 1:**
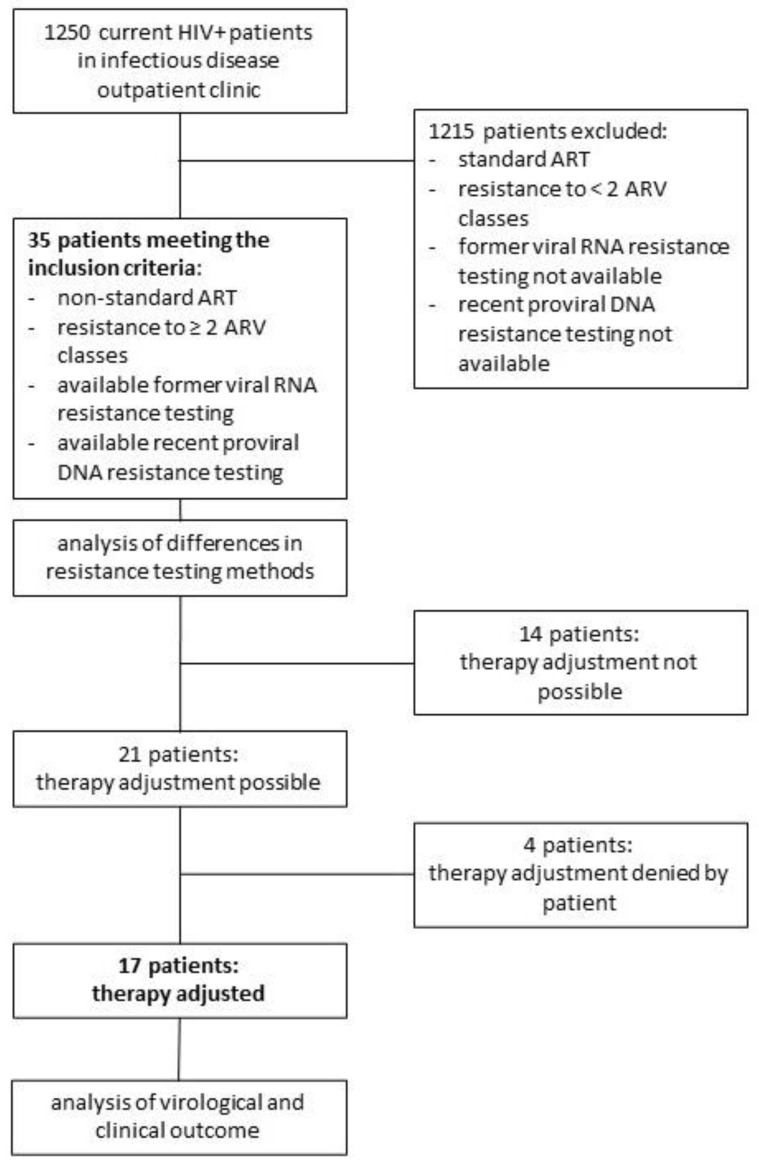
Process of patient recruitment and study design.

Between November 2017 and August 2020, 35 out of 1250 (2.8%) patients living with HIV were identified as receiving a non-standard ART regimen due to resistance to two or more ARV classes and in whom resistance tests on proviral HIV DNA templates were present. The recruitment process and study design are presented in Figure 1. Basic characteristics of all 35 patients are summarized in Table 1. The median length of HIV infection since diagnosis was 27 years (IQR 5–35), and 24 out of 35 (69%) patients were at CDC stage 3 (AIDS). The median ART duration was 22 years (IQR 5–26), and 16 out of 35 (46%) had already received all five ARV classes available during that time. The study’s patient group included various individuals, including a 26-year-old young man who had been diagnosed with HIV infection at the age of 3 years, indicating likely vertical transmission. He initiated ART at the age of 10 years. Despite achieving current stable viral suppression, there was no available information regarding ART adherence during childhood and early adolescence.

### 3.2. Analysis and Comparison of Detected Resistance Mutations in Viral RNA and Proviral DNA

Based on the data collected, it was found that a median of six RNA genotypic resistance tests were performed (IQR 2–19). In addition, a median of one proviral DNA genotypic resistance test (IQR 1–3) was conducted. The median time between the last RNA and the current genotypic resistance testing of proviral DNA was 7 years (IQR 0–17). The results revealed that the majority of patients (31/35, 89%) showed resistance to three or more ARV classes, as shown in Table 2.

#### 3.2.1. NRTI-Associated Resistance Mutations

All patients developed NRTI-associated resistance mutations over the course of their disease. Only four patients (11%) showed no resistance mutations to NRTIs in proviral DNA resistance testing. The most frequently mutated key positions in plasma were M184 (30/35, 86%) and for thymidine analogue mutations (TAMs) T215 (26/35, 74%) and M41 (23/35, 66%). In comparison, the TAMs T215 (23/35, 66%) and M41 (22/35, 63%) were detected most frequently in the proviral DNA.

#### 3.2.2. PI associated Resistance Mutations

In the cumulative history of resistance tests of viral RNA, PI-associated resistance mutations could be detected in 30 out of 35 (86%) patients. In contrast, PI-associated resistance mutations were found in the proviral DNA in only 26 out of 35 (74%) patients. The most frequently detected mutated key positions in plasma were I54 (19/35, 54%), M46 (17/35, 49%) and V82 (15/35, 43%). At the proviral level, the order of the most frequently found mutated key positions was the same: I54 (16/35, 46%), followed by M46 (14/35, 40%) and V82 (12/35, 34%).

#### 3.2.3. NNRTI-Associated Resistance Mutations

In all patients, NNRTI-associated resistance mutations could be found in past resistance tests of viral RNA. In the current resistance testing of proviral DNA, resistance to NNRTIs could be detected in only 26 out of 35 (74%) patients. The most commonly detected key positions for mutations in plasma were K103 (21/35, 60%), Y181 (18/35, 51%) and G190 (9/35, 26%). Mutated key position K103 (12/35, 34%) was also detected most frequently in the proviral DNA, followed by Y181 (8/35, 23%) and G190 (5/35, 14%).

#### 3.2.4. INI-Resistance-Associated Resistance Mutations

Only 54% of the patients had a genotypic resistance test of viral RNA for INIs. Of these nineteen patients, only six showed INI-associated resistance mutations in the cumulative history of resistance testing of viral RNA. The most commonly detected mutated key position in plasma was N155 (4/19, 21%). One patient each had a mutation at the key position E138, G140 or Q148. In the analysis of proviral DNA testing, it was shown that only one patient exhibited a resistance mutation.

#### 3.2.5. Comparison of Resistance Mutations between Viral RNA and Proviral DNA—Higher Frequency of Resistance Mutations in Viral RNA

**Figure 2:**
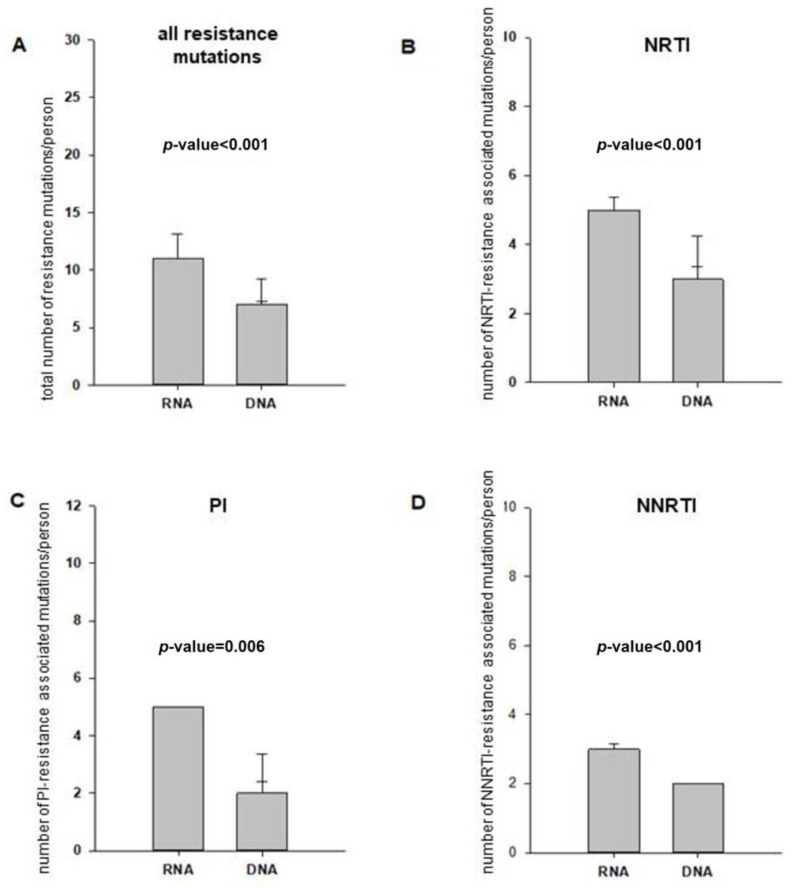
Comparison of resistance mutations found in RNA and DNA per person. (**A**) Comparison of resistance mutations found in total per person in the cumulative history of RNA GRT and found in proviral DNA GRT. The median number of resistance mutations found in the reverse transcriptase (RT) and protease (PR) gene per patient was 11 (IQR: 2–24) for resistance testing of viral RNA and 7 (IQR 1–19) for proviral DNA. Data are presented as median with standard error. *p*-value < 0.001 as determined by Wilcoxon signed-rank test. (**B**) Comparison of NRTI resistance-associated mutations found in total per person in the cumulative history of RNA GRT (median number: 5, IQR: 1–9) and found in proviral DNA GRT (median number: 3, IQR 0–8). Data are presented as median with standard error. *p*-value < 0.001 as determined by Wilcoxon signed-rank test. (**C**) Comparison of PI resistance-associated mutations found in total per person in the cumulative history of RNA GRT (median number: 5, IQR: 0–10) and found in proviral DNA GRT (median number: 2, IQR: 0–9). Data are presented as median with standard error. *p*-value = 0.006 as determined by Wilcoxon signed-rank test. (**D**) Comparison of NNRTI resistance-associated mutations found in total per person in the cumulative history of RNA GRT (median number: 3, IQR: 1–8) and found in proviral DNA GRT (median number: 2, IQR: 0–6). Data are presented as median with standard error. *p*-value < 0.001, as determined by Wilcoxon signed-rank test.

Only one patient showed concordant results in the genotypic resistance test of viral RNA and proviral DNA for all three substance classes NRTI, PI and NNRTI. Considering the ARVs individually, the same resistance profiles in RNA and proviral DNA could be demonstrated for the NRTIs in 8 out of 35 (23%) patients and for the PIs and NNRTIs in 10 out of 35 (29%) patients. Of the patients with historical resistance tests for INIs, 12 out of 19 (63%) patients showed concordant resistance profiles in the DNA. Significant differences were seen in the number of resistance mutations found in viral RNA and proviral DNA for the three substance classes of NRTIs, PIs and NNRTIs. The data indicated a higher frequency of resistance mutations in viral RNA compared to proviral DNA (as shown in Figure 2). However, a total of 23 DRMs (9 NRTI-associated, 11 PI-associated and 3 NNRTI-associated resistance mutations) were detected via proviral DNA testing that had not previously been known following RNA testing. For NRTIs and PIs, these led in part to the extension of resistance to more substances of this drug class. The median for INI-associated resistance mutations was 0 for both RNA (IQR 0–3) and DNA (IQR 0–1).

### 3.3. Virological Course after Therapy Adjustment

**Figure 3:**
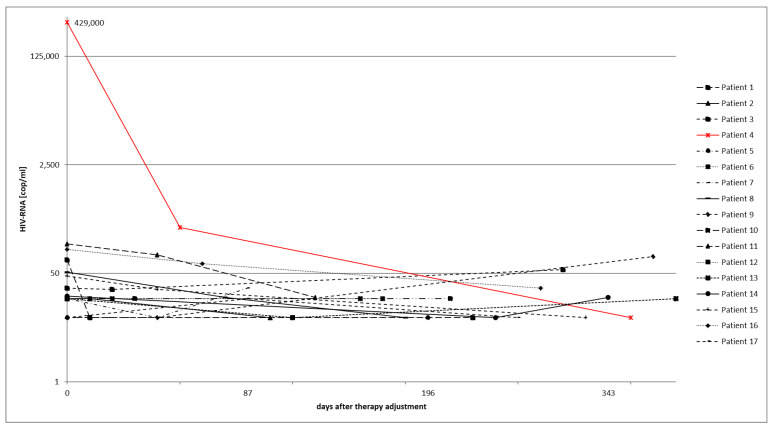
Course of HIV-RNA in patients with therapy adjustment. 0 = Time of therapy adjustment. In the logarithmic representation of the HIV RNA viral load, most patients are in the range of <50 cop/mL before and after a change in therapy. Patient 4 (red marked) had a phase of discontinuous adherence to the therapy. After adjusting the regimen with a reduction of the pill burden from 9 to 3 pills per day, the adherence was optimal, showing a viral load beneath the detection level.

ART was simplified in 17 patients based on the current results of proviral resistance testing. The median time between therapy adjustment and the first follow-up examination was 28 days, with a range of 20–251 days. The median time between therapy adjustment and the last follow-up examination was 202 days, with a range of 21–636 days. Figure 3 shows the course of viral load after adjustment. At baseline, 12 out of 17 patients (71%) had HIV RNA <50 cop/mL. However, after treatment adjustment, during the last follow-up examination, 15 out of 17 patients (88%) had HIV-RNA levels below 50 cop/mL. Furthermore, before therapy adjustment, 16 out of 17 patients (94%) had HIV-RNA levels below <200 cop/mL. In the last follow-up examination, all 17 patients had HIV RNA levels below 200 cop/mL. Of the 12 patients with HIV RNA <50 cop/mL before therapy simplification, all maintained this level at the first follow-up visit. However, only 11 of them had a viral load <50 cop/mL at the last follow-up. One patient had an increase in HIV RNA to 91 cop/mL at the last follow-up and further up to 1050 cop/mL. A file entry indicated that the patient had missed taking the medication. Before the therapy change, five patients had viral loads higher than 50 HIV cop/mL. Four of these patients had viral loads between 50 and 200 cop/mL, while one had a viral load greater than 200 cop/mL. However, following treatment simplification, all five patients experienced a decrease in viral load. In the last follow-up examination, four of the patients with HIV RNA between 50 and 200 cop/mL had viral loads lower than 50 cop/mL. Additionally, the patient with an initially high viral load above 200 cop/mL showed a very positive response to therapy, with a decrease in viral load to below 200 cop/mL. A regimen adjustment (INI/PI/NRTI) was required due to side effects, in which the NRTI component was replaced by an entry inhibitor. Despite the change, the patient’s viral load consistently remained below 50 cop/mL.

### 3.4. Reduction in Daily Pill Intake and Number of Drug Classes

**Figure 4:**
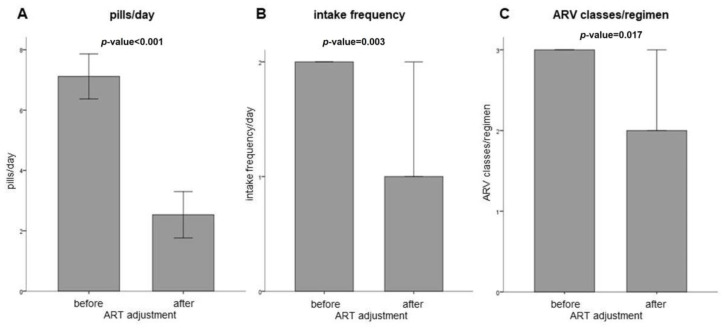
Comparison of ART regimens before and after adjustment. (**A**) Comparison of average pills intake per day before and after therapy adjustment. Data are presented as mean average with standard error. *p*-value < 0.001 as determined by paired *t*-test. (**B**) Comparison of daily intake frequency pre- and after-therapy adjustment. Data are presented as median with a 95% confidence interval. *p*-value = 0.003 as determined by Wilcoxon signed-rank test. (**C**) Comparison of ARV classes per regimen pre- and after-therapy adjustment. Data are presented as median with a 95% confidence interval. *p*-value = 0.017, determined by Wilcoxon signed-rank test.

Figure 4 illustrates that therapy simplification led to a reduction in daily pill intake in 16 patients. The mean number of pills per day decreased significantly from eight to three (*p* < 0.001). In 10 of these patients, the dosing frequency was also reduced, with the median frequency dropping from twice per day to once per day. (range before adjustment: 1–2, after 1–2; *p* = 0.003). Overall, there was a significant decrease in the number of ARV classes in the patients’ regimens from three to two (*p* = 0.017). Out of all patients, only two had more ARV classes in their new regimen compared to their old regimen.

The number of patients taking NNRTIs fell from six to one, as did it for PIs from 15 to 11. Additionally, the number of patients taking entry inhibitors was reduced from seven to two. Prior to the therapy switch, only eight patients received NRTI-containing regimens. However, following the adjustment, 11 regimens contained the NRTI class. The proportion of patients taking INIs remained relatively stable after switching therapy: prior to simplification, 11 patients were taking INIs, and this number only increased slightly to 12 after simplification. An overview of the existing ART regimens and the underlying resistance-associated mutations based on RNA testing, as well as the mutations detected in proviral DNA testing and the new ART regimens, is provided in Table 3.

## 4. Discussion

Drug resistance incidence in high-income countries has decreased but must still be considered when selecting ART for advanced HIV patients with resistance to multiple drug classes [2,3,23]. Moreover, complex ART regimens often result from resistance developed over many years, particularly during periods when treatment options were limited. In this study, most patients had started antiretroviral therapy before or just after the introduction of the first PI in 1996. Additionally, a considerable percentage of individuals (69%) had advanced HIV infections, with AIDS-defining diseases or reduced CD4 cell counts. Furthermore, all patients showed resistance to at least two but usually to at least three ARV classes (89%). Notably, resistance was predominant among the (N)NRTIs, which are among the oldest agents with a low resistance barrier [24]. As expected, the lowest resistance rates were found in the integrase inhibitor class. It is important to note that there were significantly more resistance mutations detected in the genotypic resistance analysis when using viral RNA compared to proviral DNA. In addition, a mismatch between viral RNA and proviral DNA was detected in 20% of patients in the INI group. Previous studies have shown that resistance analysis using viral RNA can detect fitter, resistant viral variants before viral suppression has occurred [25,26]. In this case, the concordance between resistance assays using viral RNA and proviral DNA is highest [11,27,28]. The ability of a viral variant to archive its genome in the cell reservoir of latent infection may also depend on how long its replication phase lasts [29]. At the same time, archived viral variants appear to change despite viral suppression without new resistance leading to an outbreak of infection, suggesting subliminal replication of fitter viruses and a possible reason for the loss of old resistance mutations over time [30,31,32]. The concordance between resistance assays tends to decrease when there are more changes in the treatment regimen and when the patient has been on ART for a longer period of time [33,34]. The concordance between proviral DNA-based and RNA-based resistance testing thus depends on multiple factors, especially concerning time and ART, within the dynamics of HIV infection [35]. In this cohort, the time between genotypic resistance analysis using viral RNA and the current analysis using proviral DNA was usually 7 years, and patients had been prescribed an average of 10 different ART regimens, possibly mapping two different viral populations in different states (uncontrolled vs. controlled viral replication).

The use of complex ART regimens is often a consequence of resistance that accrues gradually over time, typically during periods in which treatment options were limited [23]. In this study, the majority of patients had started antiretroviral therapy before or shortly after the ART era began (introduction of the first PI in the European Union in 1996), and nearly half (46%) had already taken each of the five common ART drug classes at least once during a treatment regimen. For long-term survivors who reach an older age, taking multiple medications can become challenging due to comorbidity and the risk of drug interactions that come with polypharmacy. The high number of tablets and frequency of administration with which ARVs are taken can significantly affect patients’ quality of life. As shown by Ma et al. [36], a justified, not too-frequent change of streamlined ART regimen can provide a survival advantage over rigidly maintaining an apparently effective therapy. Therefore, the offer of a modern, simplified therapy for these patients should primarily aim to minimize these risks and increase the quality of life without losing antiviral efficacy. In order to achieve this, a cohort of 17 patients underwent genotypic resistance testing of proviral DNA as an added tool in the decision-making process for their therapy. This approach led to successful treatment outcomes for the majority of patients in the cohort.

Only two cases demonstrated therapy failure: one due to a lack of adherence to therapy, leading to an increase in viral load, and another in which the regimen had to be changed due to adverse drug reactions despite continued viral suppression.

The efforts to simplify therapy have resulted in a significant reduction in both the number of tablets required and the dosing intervals for 10 patients. This is especially significant as the proportion of non-HIV-associated medication is increasing due to newly emerging comorbidities in old age as a result of a longer life span with HIV infection [37]. In particular, hidden therapies with over-the-counter drugs or age-typical drugs such as anticholinergics already carry an increased risk of side effects and interactions [38]. When considering drug interactions, it should also be noted that the age-related reduction in renal and hepatic metabolism of ARVs is well described, but studies on pharmacokinetics and pharmacodynamics in people over 60 are very limited and often rely on retrospective analyses [39]. Furthermore, a reduction in pill burden promotes patient adherence and thus also the (long-term) efficacy of ART, which is reflected in sustained viral suppression [40,41]. In particular, a gain in quality of life and, thus, increased adherence to therapy could be demonstrated for the pharmaceutical preparation as a single-tablet regimen [42]. The interdependence between quality of life and treatment adherence has been acknowledged as a critical factor for patient outcome [43]. Consequently, there is a great need for simplification and reducing the medication burden, driving the development of antiretroviral agents [44]. Notably, in our cohort, 14 decisions (70%) to adjust therapy were motivated by a patient’s desire to reduce pill burden.

While our work has yielded valuable insights, it is important to acknowledge that there are limitations. The sample size of 35 patients was relatively small, as the inclusion criteria were strict, and the number of patients with an appropriate history is small, even in a university-based specialized outpatient clinic. Of those, only 17 patients were able to undergo therapy adjustment, as others were either unsuitable for therapy adjustment due to current resistance or comorbidities or simply refused. In addition, the retrospective evaluation of real-life data that was not subject to a standardized protocol, resulted in inhomogeneous follow-up data ranging from 3 weeks to 1.75 years. In addition, the therapy changes were elective measures without an urgent medical reason, with the exception of patient 4′s adherence problems. This means that one effective therapy was to be replaced by another. Therefore, due to very low viral loads, a direct comparison of the resistance profiles from RNA and DNA was not possible, and historical findings had to be relied upon. Furthermore, in our analysis, we used a “clinical” cut-off value of 2% to identify minor variants, emphasizing the importance of comprehending both the technical and clinical aspects associated with such detection. Despite the fact that it is technically possible to detect valid variants with frequencies below 2%, there is a possibility of false-positive detections, even in treatment-naïve patients [45]. However, there is evidence that some DRMs might have an impact when present at frequencies exceeding 2% [46]. This suggests that the clinical cut-off value for interpreting the relevance of detected DRMs would likely center around 2%. As a result, in our daily practice, we focus our attention solely on mutations detected at 2% or higher. Notably, bioinformatic algorithms designed to exclude irrelevant mutations, e.g., due to APOBEC3 hypermethylation, rely on predefined variables and probabilities. While they reflect current knowledge, they may not provide complete accuracy in all cases.

Thus, it cannot be ruled out that some of the mutations found belong to proviruses without replication capability and without clinical relevance. On the other hand, the PMBCs present in the blood sample only provide an insight into a section of the entire latent infection reservoir [35]; therefore, a lack of detection of relevant viruses is also conceivable.

Nevertheless, in cases where changing an ART is being considered, current guidelines suggest taking into account various data of the patient’s history [47]. The use of proviral DNA for resistance testing can complete this information, especially in the absence of knowledge of the current resistance situation and suppressed viral load [17,18]. By offering additional insight, genotypic resistance testing using proviral DNA can help healthcare providers to make informed treatment decisions, even in complex cases involving patients who have been on multiple regimens for extended periods and to continue to maintain stable viral suppression using non-standard ART. However, therapy changes should only be made when necessary and with careful consideration of potential drug interactions and comorbidities.

## Figures and Tables

**Table 1:** Overview of the demographic, epidemiological and virological characteristics as well as the anamnestic therapy data of all 35 patients.

Characteristics (*n* = 35)	Results
**demographics**	
age, years, median (range)	60 (26–79)
female gender, %	26
ethnicity, caucasian, %	82
**comorbidity**	
coinfection with hepatitis virus (HBV, HCV), %	40
chronic kidney disease, %	9
atherosclerotic vascular diesases, %	26
arterial hypertension, %	40
diabetes mellitus, %	11
psychiatric diagnosis, %	29
**HIV parameters**	
years of HIV infection, median (range)	27 (5–35)
CDC stage 3, %	69
HIV subtype B, %	69
R5 tropism, %	45
current CD4 count, cells/µL, median (range)	500 (100–1370)
current CD4 count, %, median (range)	23.55 (9–48)
Nadir CD4 count, cells/µL, median (range)	110 (0–440)
maximum viral load, cop/mL, median (range)	308,956 (13,500–6,822,000)
**antiretroviral therapy data**	
duration of ART, years, median (range)	22 (5–26)
history of mono/dual therapy, %	49
number of ART regimens, mean (range)	10 (2–18)
5 ARV classes in history, %	46
number of ARV classes in current regime, mean (range)	3 (1–4)
years of viral suppression, median (range)	8 (0–16)

HBV = hepatitis B virus; HCV = hepatitis C virus; CDC = centres of disease control; ART = antiretroviral therapy; ARV = antiretroviral.

**Table 2:** Overview of the general resistance situation and resistance tests in all 35 patients.

Characteristics (*n* = 35)	Results
**resistance situation**	
number of RNA GT, median (range)	6 (2–19)
number of DNA GT, median (range)	1 (1–3)
historical INI GT, %	55
resistance ≥ 2 ARV classes, %	100
resistance ≥ 3 ARV classes, %	88, 57
years between last RNA GT and current DNA GT, median (range)	7 (0–17)

GT = genotypic testing; INI = integrase inhibitor; ARV = antiretroviral. For this retrospective analysis, we only took in account the five usual ARV classes (NRTI, NNRTI, INST, PI and Entry Inhibitors) of the study period.

**Table 3:** Synopsis of all known resistance-associated mutations detected by RNA testing and the old ART regime as well as current mutations based on proviral DNA testing with new ART regimen of the 17 patients who underwent ART adjustment. Proviral DNA testing was done under (efficient) ART, while RNA testing had, in general, been performed in therapy naïve patients or in case of therapy failure. (Additional information concerning the detected resistance-associated mutations of the other 18 patients who did not undergo ART adjustment is presented in the Supplementary Material, Table S1).

Patient	NRTI-Associated Resistance Mutation (RNA)	NNRTI-Associated Resistance Mutation (RNA)	PI-Associated Resistance Mutation (RNA)	INI-Associated Resistance Mutation (RNA)	Old ART Regimen	NRTI-Associated Resistance Mutation (DNA)	NNRTI-Associated Resistance Mutation (DNA)	PI-Associated Resistance Mutation (DNA)	INI-Associated Resistance Mutation (DNA)	New ART Regimen
**1**	L74V, M184V, T215Y, M41L, L210W, K219E/N, D67N,	G190S, K101E, Y181C,	V82T, I54V, L24I, M46I, G73S, Q58E	na	**FPV + rtv + AZT + TDF + MVC**	L74V, M184V, T215Y, M41L, L210W, K219N	Y181C, G190S, K101H/Q	V82S/T, I54V, L24I, M46I, G73S, L33F	-	**TAF/FTC/EVG/cob + DRV** Due to adverse drug effects switched to: **DRV + rtv + DTG + MVC**
**2**	M184V, T215F, M41L	K103N	I54V, V82A	na	**TAF/FTC + DRV + rtv + MVC**	-	-	-	-	**TAF/FTC/DRV/cob**
**3**	M184V, T215Y, M41L, L210W	H221Y, K103N	V82L	na	**LPV/rtv + RAL**	M184V, T215N/S/Y, L74V, M184I/V, T215N/S/Y, M41I/L, L210W M41L	K103N	V82L	-	**BIC/TAF/FTC + MVC**
**4**	M184V, L74I	V106I, Y181C, E138A/P/Q	T74P, K20T, L33F, I84V, V82I/M,	-	**TDF + ETR + RAL + DRV + rtv**	M184V, K219N	Y181C E138K	-	-	**DRV+ rtv + DTG**
**5**	L74V/I, M184I/V, T215C/Y, M41L, D67G, K70R, K219E, V75A	Y188F/H, L100V, V106I, K103N, A98G, V108I, H221Y, Y318F	V82T, L90M, I54V, G73S, G48A/M/T/V, F53L	-	**RAL + MVC + rtv + DRV + 3TC/ABC**	L74I, M184V, T215Y, M41L, D67G/N/S, K70R, K219E	Y188F/H/L, A98G, V108I, H221Y, L100V, V106I	V82T, L90M, I54V, G73S	-	**DRV + rtv + RAL**
**6**	L74V, K70R/N, T215N/S/Y, M41L, L210W, K219R, T69D	L100I, K103N, K238T	I84V, L90M, M46I, G73S/T	na	**DRV + rtv + RAL + ETR**	L74V, M184V, T215Y, M41L, L210W, K219R, T69D	L100I, K103N	I84V, L90M, I54V, M46I, G73T, L10F	-	**TAF/FTC/DRV/cob + DTG**
**7**	T215F, K219E, M184V, D67N, K70R, M41L,	K103N, Y181C, V108I	L90M, V32I, I54V, V82A, M46I, I47V, F53L, L33F	na	**DRV + rtv** **DDI + RAL**	M184V, T215F, M41L, D67N, K70R, K219E	K103N, Y181C	L90M, V32I, I54L/V, V82A, M46I, I47V, F53L, L33F	-	**MVC + DTG/3TC**
**8**	L74V, M184V, T215F/I/S, D67N, K70G/R/S, M41L, K219H/Q, V75M, F77L	K103N, Y181C, V108I, H221Y, K101H	I84V, L24I, M46I, V32I, I54L, T74P, L89M/V, K20T, L33F, K43T	-	**RAL + DOR + DRV + rtv**	M184V, T215F/I/S, M41I/L, D67N, K70R, V75I/M, F77L, K219Q	K103N, K101H/N/Q	I84V, L24I, M46I	-	**RAL + DRV+ rtv**
**9**	M184V	K103N	-	na	**3TC/ABC + DRV + rtv + MVC**	-	E138A	-	-	**TAF/FTC/DRV/cob**
**10**	M184V, D67N, K70R, K219E	Y181C	I54V, V82A, L24I, M46L, F53L, L33F	-	**DRV + rtv + RAL + ETR**	K65R, M184V, D67N	Y181C	I54V, V82A, L24I, M46L, F53L, L33F, G73S	-	**TAF/FTC/DRV/cob + DTG + ETR**
**11**	L74V, T215Y, M41L, M184V	K103N, G190A, Y181C, H221Y, K101H	I54V/M, V82A, M46I, I84V, Q58E, L33F	na	**DRV + rtv + TDF/FTC + MVC**	-	-	-	-	**BIC/TAF/FTC**
**12**	L74V, M184V, T215Y, M41L, D67N, L210W, V75M, E44D	K103N, G190A, Y181C	I84V, L90M, M46I, L24F, L33F, G73S, I54L, L10F	-	**DRV + rtv + RAL + MVC**	M184V, T215N/S/Y, M41L, D67N, K70R, L210W	-	-	-	**DRV + rtv + DTG**
**13**	M184I/V	K103N, P225H	-	N155H	**DRV + rtv + ETR + DTG**	M184I	-	-	-	**BIC/TAF/FTC**
**14**	K65R, M184V	G190S, Y181C, K101E, A98G, E138A	L89M/V	-	**DTG + DRV + rtv**	M184V	G190S, K101E	-	-	**TAF/FTC/DRV/cob + DTG**
**15**	L74V, M41L, D67N, L210W, T215D, L74V, M41L, D67N, L210W, T215D, V75M	K103N, G190A, Y181C	I84V, L90M, I54L, M46I, L33F, L10F	-	**3TC + RAL + DRV + rtv + ETR + MVC**	L74V, M41L, D67N, L210W, T215D, V75M	K103N, G190A, Y181C	I84V, L90M, I54L, M46I, G73S, L33F, L10F	-	**TAF/FTC/DRV/cob + DTG**
**16**	D67N, T215E, K219Q	Y188L	I54L	-	**DRV + rtv + RAL + 3TC**	D67N, T215E, K219Q	Y188L	I54L	-	**BIC/TAF/FTC**
**17**	K70E/G/R	K101E	M46L	na	**RAL + DRV + rtv**	K70G	M46L	-	-	**BIC/TAF/FTC**

Ingredients are separated by “/”, formulations by “+”. na, no test result available. 3TC, Lamivudine; ABC, Abacavir; AZT, Zidovudine; BIC, Bictegravir; cob, Cobicistat; DDI, Didanosine; DOR, Doravirine; DRV, Darunavir, DTG, Dolutegravir; ETR, Etravirine; EVG, Elvitegravir; FPV, Fosamprenavir; FTC, Emtricitabine; LPV, Lopinavir; MVC, Maraviroc; RAL, Raltegravir; rtv, Ritonavir; TDF, Tenofovirdisoproxil, TAF, Tenofoviralafenamide.

## Data Availability

Raw data were generated at the University Hospital of Cologne. Derived data supporting the findings of this study are available from the corresponding author upon request.

## Supplementary Materials

The following supporting information can be downloaded at: https://www.mdpi.com/article/10.3390/v15071444/s1, Table S1: Synopsis of all known resistance-associated mutations detected by RNA testing and the current ART regime as well as current mutations based on proviral DNA testing of the 18 patients who did not undergo ART adjustment.
