# Supplementary material for: Optimizing Antiretroviral Therapy in Heavily ART-Experienced Patients with Multi-Class Resistant HIV-1 Using Proviral DNA Genotypic Resistance Testing
Source: Viruses. 2023 Jun 27;15(7):1444. doi: 10.3390/v15071444 (PMC10384096; doi:10.3390/v15071444)
Supplement: Supplementary file 1 [file viruses-15-01444-s001.zip › viruses-2455512-supplementary.pdf]

Table S1: Synopsis of all known resistance associated mutations detected by RNA testing and the current ART regime as well as current mutations based on proviral DNA testing of the 18 patients who did not undergo ART adjustment. Proviral DNA testing was done under efficient ART, while RNA testing had in general been performed in therapy naïve patients or in case of therapy failure. Ingredients are separated by "/", formulations by "+". na, no test result available.

patient	NRTI-associated resistance mutation (RNA)	NNRTI-associated resistance mutation (RNA)	PI-associated resistance mutation (RNA)	INI-associated resistance mutation (RNA)	current ART regimen	NRTI-associated resistance mutation (DNA)	NNRTI-associated resistance mutation (DNA)	PI-associated resistance mutation (DNA)	INI-associated resistance mutation (DNA)
**18**	K65R, M184V	K103N, Y181C, H221Y	I84V, I54M, L33F, L10F	-	**RAL + DRV + rtv**	-	K103N, Y181C, H221Y	L10F, L33F, I54M, I84V	-
**19**	L74ILV, T215F/I/S, M41L, D67N, K70R, V75A/I/T, L210W, K219Q, M184V	G190A, Y181C, K101E	L90M, V82A, G73C, L33F, I50V, I54A/V, F53L, T74P, K43T, M46I	-	**DRV + RAL + DOR + TAF/FTC + rtv**	L74V, M184V, T215F/I/S, M41L, D67N, K70R, K219Q	G190A, Y181C, K101E	L90M, I54V, V82A, M46I, F53L, G73C, L33F, I50V, K43T	-
**20**	T215Y, D67N, K70R, K219E, M184V	Y181C	I47A, V32I, V82A, I54L, L76V, L89V	-	**DRV + MVC + ETR + rtv**	T215Y, D67N, K70R, K219E	Y181C	V82A/C/G/S, L90M, V32I, I54L, M46I, L76V, L10F/I, L89V	-
**21**	M184V, T215F/A/S, M41L, T69D, L74V, D67N, K70R, L210W, K219Q	L100I, K103N, V179I/L/P/T, E138G, Y181I	L90M, I54V, V82A, G48A/M/T/V, F53L, N83D, L33F, K43T, M46L, I84V	N155H	**DTG/3TC + DRV + cob**	M41L, D67N, L210W, T215S, K219Q, T69D	E138G	I54V, N83D, L33F	-
**22**	L74V/A/I, K70G/R, M184V, T215Y, V75A	K103N, V108I, H221Y, E138A	N88S, L90M	na	**DTG + DRV + rtv**	-	-	-	-
**23**	M184V, T215N/S/Y, M41L, K70R	M230I	L33F	E138A	**FTC/TAF/DRV/Cob + DTG**	M184I, T215N/S/Y, M41I/L	-	G73S	-
**24**	M184V, T215C/Y, M41L, L210W, L74V	L100I, K103N	I47A, M46I	na	**DRV + DTG + rtv**	-	-	-	-
**25**	M184I	E138K	-	na	**TAF/FTC/EVG/cob + DRV**	-	-	-	-
**26**	L74I/V, T215Y, M41L, D67E/G, L210W, K219N, M184V	K103N, G190A, Y181C, K238T, H221Y	I54V, V82T/A, L24M/I, M46I, L10F/I, K43T, I84V	N155H	**ETR + DRV + TAF/FTC + rtv**	L74I/V, M184V, T215N/S/Y, M41L, D67G, L210W, K219N	K103N, Y181C, K238T	I84V, V82A/T, I54V, L24I, M46I, L10F/V, K43T	-
**27**	T215Y, M41L, D67N, K70R, L210W, K219E, M184V	Y318F, V108I, Y181C, H221Y, K103N	I54A/S/V, V82A , M46I, L33F, K43T, L90M	na	**DTG + DRV + rtv**	M184V, T215N/S/Y, M41L, D67N, K70R, L210W, K219D/E/N	Y318F, V108I, Y181C, H221Y, K103N	L90M, I54F/S/T, V82A, M46I, L33F, K43T	-
**28**	L74I, M184V, T215Y, M41L, D67N, L210W	L100I, K103N, A98G, E138G	-	Q148H, G140S	**MVC + DRV + rtv**	M184I/V, T215N/S/Y, M41I/L, D67N, L210W, K219R, E44A	K103N, A98G	G73S	-
**29**	M184V, T215C/Y, M41L, L210W	K238T	-	na	**RAL + DRV + rtv**	M184V, T215C/Y, M41L, L210W	K238T	M46L	-
**30**	L74V, M184V, T215F, M41L, D67N, L210W, K219E	L100I, K103N	G48V, L90M, V82A/S, I54T, F53L	na	**RAL + DRV + rtv**	L74V, M184V, T215F, M41L, D67N, L210W, K219E	L100I, K103N	G48V, I54T, V82A	-
**31**	M41L, T215A/D/N/Y, M184V	K103N, Y181C, F227L	L90M, V32I, M46I, I47V, L89V, K43T	na	**TDF/FTC + ETR + RAL + DRV + rtv**	T215D, M41L, M184V	-	-	-
**32**	T69Ins, T215Y, M41L, A62V, D67E, L210W	Y188F/L, Y181D/V	N88D, L90M	-	**DRV + DTG + MVC + rtv**	T215Y, M41L, A62V	-	N88D	-
**33**	M184V, D67N, K70R, K219Q	G190A, A98G	G73A	N155H, D232N, G118R	**DRV + rtv**	-	A98G	-	-
**34**	L74V, M184V, T215F , M41L , L210W, K70R, K219Q	L100I, K103N	V82T/S, I54V, L24I, L33F, M46L, K43T	na	**RAL + ETR + DRV + rtv**	M184V, M41L	-	V82A/F/S, I54V	-
**35**	T69Ins, T215C/Y, M41L, L210W, V75M, M184V	V106I/M, G190A, Y181C, K101H	I84V, Q58E, M46I, L90M, L10F, L89V, K20T, I54A, V32I, G73D	-	**TAF/FTC + DTG + MVC**	T69Ins, M41L, V75M	-	I84V, L90M, I54A, L10FY, L89V, Q58E	-

3TC, Lamivudine; ABC, Abacavir; AZT, Zidovudine; BIC, Bictegravir; cob, Cobicistat; DDI, Didanosine; DOR, Doravirine; DRV, Darunavir, DTG, Dolutegravir; ETR, Etravirine; EVG, Elvitegravir; FPV, Fosamprenavir; FTC, Emtricitabine; LPV, Lopinavir; MVC, Maraviroc; RAL, Raltegravir; rtv, Ritonavir; TDF, Tenofovirdisoproxil, TAF, Tenofoviralafenamide.
